# Effects of a partially supervised conditioning programme in cystic fibrosis: an international multi-centre randomised controlled trial (ACTIVATE-CF): study protocol

**DOI:** 10.1186/s12890-018-0596-6

**Published:** 2018-02-08

**Authors:** Helge Hebestreit, Larry C. Lands, Nancy Alarie, Jonathan Schaeff, Chantal Karila, David M. Orenstein, Don S. Urquhart, Erik H. J. Hulzebos, Lothar Stein, Christian Schindler, Susi Kriemler, Thomas Radtke, the ACTIVATE-CF Study Working Group

**Affiliations:** 1 Paediatric Department, Julius-Maximilians University, Josef-Schneider-Str. 2, 97080 Würzburg, Germany; 2 Montreal Children’s Hospital, McGill University Health Centre, Montreal, Quebec Canada; 3 Service de Pneumologie et Allergologie pédiatriques, Centre de Ressources et Compétences dans la Mucoviscidose, Hôpital Necker Enfants Malades, Université Paris V – Descartes, Paris, France; 4 Department of Pediatrics, Children’s Hospital of Pittsburgh of UPMC, Pittsburgh, USA; 5 Department of Paediatric Respiratory and Sleep Medicine, Royal Hospital for Sick Children, Edinburgh, UK; 6 Child Development & Exercise Center, Wilhelmina Children’s Hospital, University Medical Center Utrecht, Utrecht, The Netherlands; 7 Hannover Medical School, Institute of Sports Medicine, Hannover, Germany; 8 Swiss Tropical and Public Health Institute, University of Basel, Basel, Switzerland; 9 Epidemiology, Biostatistics and Prevention Institute, University of Zurich, Zurich, Switzerland

**Keywords:** Physical activity, Exercise intervention, Randomised controlled trial, Partially-supervised, Cystic fibrosis

## Abstract

**Background:**

Physical activity (PA) and exercise have become an accepted and valued component of cystic fibrosis (CF) care. Regular PA and exercise can positively impact pulmonary function, improve physical fitness, and enhance health-related quality of life (HRQoL). However, motivating people to be more active is challenging. Supervised exercise programs are expensive and labour intensive, and adherence falls off significantly once supervision ends. Unsupervised or partially supervised programs are less costly and more flexible, but compliance can be more problematic. The primary objective of this study is to evaluate the effects of a partially supervised exercise intervention along with regular motivation on forced expiratory volume in 1 s (FEV_1_) at 6 months in a large international group of CF patients. Secondary endpoints include patient reported HRQoL, as well as levels of anxiety and depression, and control of blood sugar.

**Methods/design:**

It is planned that a total of 292 patients with CF 12 years and older with a FEV_1_ ≥ 35% predicted shall be randomised. Following baseline assessments (2 visits) patients are randomised into an intervention and a control group. Thereafter, they will be seen every 3 months for assessments in their centre for one year (4 follow-up visits). Along with individual counselling to increase vigorous PA by at least 3 h per week on each clinic visit, the intervention group documents daily PA and inactivity time and receives a step counter to record their progress within a web-based diary. They also receive monthly phone calls from the study staff during the first 6 months of the study. After 6 months, they continue with the step counter and web-based programme for a further 6 months. The control group receives standard care and keeps their PA level constant during the study period. Thereafter, they receive the intervention as well.

**Discussion:**

This is the first large, international multi-centre study to investigate the effects of a PA intervention in CF with motivational feedback on several health outcomes using modern technology. Should this relatively simple programme prove successful, it will be made available on a wider scale internationally.

**Trial registration:**

ClinicalTrials.gov Identifier: NCT01744561; Registration date: December 6, 2012.

## Background

Regular physical activity (PA) and exercise have become an accepted and valued part of cystic fibrosis (CF) care [1–3]. Two supervised exercise intervention studies have proven that regular vigorous exercise can positively impact forced expiratory volume in 1-s (FEV_1_) [4, 5], the single best prognostic factor for CF [6]. It has been suggested that the positive effects of regular exercise on pulmonary function might be moderated by improved airway clearance from secretions [1] and strengthening of respiratory muscles [7]. However, the exact mechanisms are unclear.

Supervised exercise interventions are expensive, not easily implemented in regular patient care and long-term adherence to supervised programs is often low [8]. In particular, long-term adherence following supervised exercise programs can be problematic [7] as such programs are often relatively monotonous and do not respond to the individual preferences of participants. Involving patients in decisions making around their exercise programs may overcome this barrier to sustainability [9, 10].

Exercise programs which are home-based and unsupervised or partially supervised have several advantages over supervised programs [7]: i) the programme can be easily implemented; ii) the patients can choose activities based on their personal preferences; iii) the accessibility of the activities with respect to location and time is much greater; iv) physical activities can be planned together with family members or friends; and v) the costs per patient and travel time commitments are much lower compared to a supervised programme. These advantages may enhance long-term positive changes in lifestyle not seen in standard supervised exercise programs [11]. Hebestreit et al., have shown that a partially supervised exercise intervention can improve forced vital capacity (FVC), exercise capacity and health-related quality of life (HRQoL) in CF [7]. Furthermore, this study demonstrated lasting benefits for these variables at 18 and 24 months after the end of the intervention [7]. If the benefits of such a partially supervised intervention can be applied to a wider population of CF patients with different health beliefs who are cared for by different health care systems, this would have significant implications for long-term outcomes in CF patients.

This randomised, parallel group study evaluates our hypothesis that a partially supervised PA programme paired with motivational feedback aimed at increasing vigorous habitual PA by at least 3 hours per week, can improve FEV_1_ and that this can be sustained over the subsequent six-month timeframe.

## Methods

### Study design

**Fig. 1:**
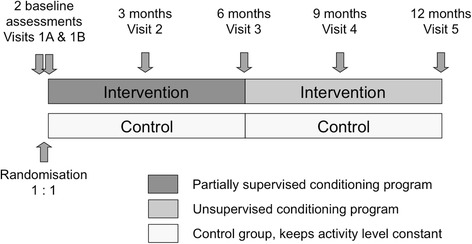
Study design and assessment time points

This study is an international multi-centre, randomised controlled trial with a parallel group design (Clinical trials.gov Identifier: NCT01744561). The study is conducted across CF centers in eight countries (Austria, Canada, France, Germany, Netherlands, Switzerland, United Kingdom and the United States). Patients are randomly allocated to either a partially supervised PA intervention or control intervention (no PA), see Fig. 1. Due to the nature of the intervention, blinding of participants is not possible. However, blinding of care team members and controls with respect to measurement results are done whenever possible. The study is conducted in full conformance with the principles of the “Declaration of Helsinki” (as amended in Tokyo, Venice and Hong Kong) and with the EC/ICH-Guidelines on Good Clinical Practice. The study is restricted to patients who provided written informed consent.

### Study population

To be eligible for enrolment, patients with CF from all participating countries and centres who meet all inclusion criteria will be invited to participate in the study: i) Confirmed diagnosis of CF based on either two CF-causing mutations and/or a sweat chloride concentration during two tests of > 60 mmol/l; ii) age ≥ 12 years; iii) FEV_1_ ≥ 35% predicted [12]; iv) Access to the internet. Patients are excluded from the study if they have at least one of the following exclusion criteria: i) Participation in another clinical trial up to 4 weeks prior to the first baseline visit; ii) Status post lung transplantation; iii) Pregnancy/Breastfeeding; iv) Inability to exercise; v) More than 4 h of reported vigorous PA’s per week currently or up to 3 months prior to baseline measurements and not already planned within the coming 6 months; vi) Unstable condition affecting pulmonary function or exercise participation (i.e., major haemoptysis or pneumothorax within the last 3 months, acute exacerbation and iv-antibiotics during the last 4 weeks, unstable allergic bronchopulmonary aspergillosis, planned surgery, listed for lung transplantation, major musculoskeletal injuries such as fractures or sprains during the last 2 months, others according to the impression of the medical doctor); vii) Cardiac arrhythmias with exercise; viii) Requiring additional oxygen with exercise; ix) Recent diagnosis of diabetes 3 months prior to screening or at screening; x) Recent changes in medication 1 month prior to screening (steroids, ibuprofen, inhaled antibiotics, mannitol, DNAse, hypertonic saline); xi) At least one G551D mutation and not on ivacaftor (VX770) yet but planning to start the drug or planning to stop ivacaftor and xii) Colonisation with Burkholderia cenocepacia.

### Randomisation

Participants are randomly allocated to the intervention and control group in a one-to-one ratio using block-randomisation (block sizes of 4) and stratified by country and according to whether FEV_1_ is below or ≥70% predicted (moderate/severe lung disease vs. normal/mild lung disease) [12]. A person not involved in the study created a computer-generated randomisation list that was implemented into the database (REDCap, Research Electronic Data Capture) by a person from the Clinical Trials Unit Bern, Switzerland, offering administrative database support. Randomisation is done at each study site within the database (REDCap Software-Version 5.9.2 – © 2014 Vanderbilt University Belgium) allowing complete allocation concealment. The participant’s mean FEV_1_ value in % predicted from both baseline visits (1a and 1b) is entered and a group allocation is returned automatically. We implemented a training database with identical electronic case report forms (eCRF’s) and data collection instruments to allow study site investigators to familiarise themselves with the database before entering real patient data. The database allows entering patient data on-line from different study sites into eCRF’s. As lung function and clinical status are fundamental in this study and may change within short time periods, the intervention starts immediately after randomisation has taken place.

### Withdrawal from study

The trial as a whole will be terminated prematurely if one or more serious adverse events invalidate the earlier positive benefit-risk assessment or when the time schedule cannot be met (e.g., more than doubling of recruitment time). All withdrawals are documented and the reason for withdrawal is recorded. All withdrawals will be discussed in the final report of the study. No attempt is to replace patients who withdraw from the trial.

### Physical activity intervention

#### Intervention arm

Participants in the intervention group are asked to add at least 3 hours of vigorous PA’s per week to their baseline activity. For the purposes of this study, vigorous PA is defined as activity that results in the participant breathing rapidly, only able to speak in short phrases, where heart rate is increased substantially, and sweating is present [13]. Including the activities already present at the baseline assessment, this should include at least 30 min of strength building exercises and 2 hours of aerobic PA’s per week. The remaining time can be attributed according to the participant‘s preferences. PA bouts lasting 20 min or longer will be counted with respect to total weekly training time.

At the second baseline visit, following randomisation, the participants in the intervention group will undergo an assessment of their preferences and motivation, followed by counselling immediately and at all subsequent study visits scheduled every 3 months. The counselling and activity assessments are done in each centre by a designated team member (physiotherapist or exercise specialist) who receives prior training so that a standardised approach is ensured. The participants complete a self-administered activity questionnaire and a structured interview using a standardised motivational interview form is carried out to assess the participant’s likes and dislikes with respect to PA’s and to identify possible barriers and determine the motivation (using a 10-point Likert Scale) of the participant to start specific activities and to make individualised PA recommendations. Based on the information from the questionnaire and interview, the physiotherapist/exercise specialist and the participant/his guardians engage in a dialogue identifying additional PA’s that are strength building and/or promoting aerobic fitness that will result in an additional 3 h per week of these activities at a vigorous level. Participants of the intervention group are also told a target heart rate range corresponding to 60–80% of peak oxygen uptake (VO_2peak_) for vigorous continuous aerobic type activities [13]. VO_2peak_ is determined during incremental exercise tests at baseline and after 6 months. Patients also receive training how to determine heart rate during exercise.

Participants receive further motivation by being equipped with a three-axial pedometer (Omron HJ-322 U-E). Participants are instructed to wear the pedometer on a daily basis and they are asked to complete a web-based log (http://www.activate-cf.org/) on a daily basis. The user interface of the website is programmed to allow access from classical personal computers, palm tops, tablets and smartphones. Participants are asked to document daily steps from their pedometer and training activities in their web-based logbook, using an individual pseudonym login that is provided by their study centre. In the web-based log book, data on i) vigorous PA’s performed that day (type of PA, duration), ii) total daily step count and exercise step count (steps with estimated energy expenditure of at least 3 metabolic exchange units-METS), and iii) time in sedentary activities (i.e. watching TV, playing video games without activity components, computer time, driving, taking a nap/sleeping) are entered.

The central monitoring unit in Wuerzburg, Germany is responsible for monitoring data entries on the web-based log. The participant’s CF centre will be notified if there have been no completed entries into the system for the previous 3 days or longer. The participants receive a “reminder” message on his or her mobile phone or via email if no entries have been made for three consecutive days. If there are no entries for a whole week, participants are contacted by phone from their centre’s designated activity contact person (i.e., physiotherapist, exercise specialist).

Participants complete a PA questionnaire at all clinic visits. The web-based training logs are checked at the clinic visits and discussed with the participant by the physiotherapists/exercise specialist. The physiotherapist/exercise specialist utilises this information, along with the web-based logs and the interview to provide exercise counselling at every visit to the centre. If necessary, a new activity plan will be created.

There are scheduled phone contacts after 1, 2, 4 and 5 months by the physiotherapist/exercise specialist into the training to discuss progress and to re-counsel the participants PA plan. During the monthly scheduled telephone contacts, the participant is asked about his or her weekly exercise activities and the written activity agreement is reviewed. Likewise, the web-based activity log is discussed with the participant. If one or more goals of the plan have not been met or problems have been encountered or new activity ideas come up, the plan is modified. The structured interview forms available for the initial counselling are used to assist as needed. In case of admission to the hospital, physical exercise is included in the in-patient treatment (the treating physician needs to decide what is possible).

After the six-month assessment, participants of the intervention group will keep their pedometers and still have access to the web-system to report and view their PA data. Centre staff meets with participants as part of routine care and as deemed necessary by the treating physician. Staff members do not receive the data collected, unless clinically relevant, such as a change in glycaemic control, and advise the patient based on the information that they gather from the patient.

### Control arm

The control group is instructed to keep their PA level constant over the 12 months of the study. Controls do not get informed about their fitness level during the exercise test nor receive any interpretation of the test results unless a finding is detected which requires medical attention. They are not be given an evaluation of their answers to the activity questionnaires nor on their pedometry (or accelerometry) data. At the closeout visit after 12 months, controls receive a pedometer and access to the web-based log and get a PA counselling as the intervention group had received at baseline.

### Recruitment and study procedures

This RCT is currently ongoing. The first patient was enrolled on 16th July 2014 and the estimated study completion date is December 2018.

At enrolment, a clinical assessment is performed for all patients using standardised procedures. Table 1 provides an overview about all study visits and assessments throughout the study. Two baseline visits (1b & 1b) are scheduled to evaluate inclusion and exclusion criteria. Following randomisation on baseline visit 1b, participants are seen every 3-months during the 12-month study period (4 follow-up visits, see Fig. 1).

**Table 1:** Data collected at baseline and follow-up

	Baseline		3 month	6 month	9 month	12 month
Visit	1a	1b	2	3	4	5
Time	−21 d (±7 d)	Day 1	90 d (±14 d)^a^	180 d (±14 d)^a^	270 d (±14 d)^a^	360 d (±14 d)^a^
Informed consent	X					
Medical history	X					
In−/exclusion criteria	X	X				
Physical examination, vital signs	X	X	X	X	X	X
Physical (in)activity history	X					
Anthropometry	X	X	X	X	X	X
Spirometry	X	X	X	X	X	X
Body plethysmography	X	X	X	X	X	X
Oral glucose tolerance test		X			X	
Cardiopulmonary exercise testing	X			X		X
Pedometry		X	(X)^b^	X	(X)^b^	X
Physical activity questionnaire (7-day recall)	X	X	X	X	X	X
Exacerbations, upper respiratory tract infections, antibiotic use, adverse events	X	X	X	X	X	X
CFQ-R questionnaire	X			X		X
DASS questionnaire	X			X		X
EMI 2 questionnaire	X			X		X
Diary, 3-months retrospective questionnaire, Interview^c^, Patient-initiated contact	X	X	X	X	X	X
For Intervention group only						
Exercise motivation interview^c^	X	X	X	X	X	X
Web-based diary, paper diary, pedometer^d^	XXXXXX					
Schedule of measurements for the sub-studies in selected centers						
	Baseline		3 month	6 month	9 month	12 month
Visit	1a	1b	2	3	4	5
Time	−21 d (±7 d)	Day 1	90 d (±14 d)	180 d (±14 d)	270 d (±14 d)	360 d (±14 d)
Accelerometry^e^		X		X		X
Multiple breath washout^e^	X	X		X		X
DXA^e^	X			X		X
Scintigraphy^e^	X			X		

*CFQ-R* Cystic Fibrosis Questionnaire-revised, *DASS* Depression and Anxiety Stress Scales, *DXA* Dual energy X-ray absorptiometry, *EMI* Exercise Motivational Inventory

^a^In case of an exacerbation the visit will be postponed until 14 days after oral or intravenous antibiotic treatment of exacerbation has been stopped

^b^Intervention group only

^c^At each visit and at scheduled telephone contacts

^d^Over the whole intervention period of 12 months

^e^In selected centres only

### Study endpoints and measurements

The primary outcome measure is the difference between the changes in FEV_1_ (in % predicted) from baseline (using the average of the two baseline measurements) to 6 months in the intervention group compared to controls. Secondary outcomes include change in aerobic exercise capacity (VO_2_peak), PA, HRQoL and others listed in Table 2. Vital signs, anthropometric characteristics (weight, height, skinfolds) are measured at each study visit using standardised procedures.

**Table 2:** Primary, secondary and explorative outcome measures

Outcome	Variable	Measurement
Primary endpoint		
• Lung function	FEV_1_% predicted	Spirometry
Secondary endpoints		
• Exercise capacity	VO_2peak_ % predicted	CPET
	Watt_max_ % predicted	CPET
• Physical activity	Steps per day	Pedometry
	Exercise steps per day	Pedometry
	Reported physical activity (min per day)	7-day recall questionnaire
• Pulmonary function	FEV_1_% predicted	Spirometry, Bodyplethysmography
	FVC % predicted	
	RV/TLC %	
• Exacerbation	Time to first exacerbation	Questionnaire (modified Fuchs criteria) and physician assessment
• Body composition	Body fat	Skinfolds
	Lean body mass	Skinfolds
• Health-related quality of life	Scales	Questionnaire (CFQ-R)
• Depression, anxiety and stress scales	Scales	Questionnaire (DASS)
• Glycaemic control	Plasma glucose concentrations (fasting, 1 and 2 h after standardised glucose load)	Oral glucose tolerance test^a^
Explorative endpoints		
• Physical activity	Moderate and vigorous physical activity	Accelerometry^b^
• Infections	Number of upper respiratory tract infections	3-months retrospective questionnaire, Interviews, Diary and telephone calls
• Antibiotic therapy	Days on additional oral/intravenous antibiotics	3-months retrospective questionnaire, Interviews, Diary and telephone calls
• Body composition	Body mass index	Based on measured height and weight
• Lung function	Lung clearance index	Multiple breath washout^c^
• Mucociliary clearance	Mucociliary clearance	Scintigraphy^d^
Safety endpoints		
• Adverse events	Adverse events at least possibly related to exercise (e.g., sprains, fractures etc)	Judged by investigator
Others		
• Compliance with exercise	Exercise goal of 3 additional hours of vigorous activity per week	Baseline assessment and web-based diary

*CFQ-R* Cystic Fibrosis Questionnaire-revised, *CPET* cardiopulmonary exercise test, *FEV*_*1*_ forced expiratory volume in 1 s, *FVC* forced vital capacity, *RV/TLC* residual volume/total lung capacity, *VO*_*2peak*_ peak oxygen uptake, *Watt*_*max*_ maximal aerobic power

^a^only in patients without cystic fibrosis related diabetes

^b^only in European centers

^c^selected centers only

^d^US centers only

#### Pulmonary function

Spirometry and body plethysmography is performed according to ERS and ATS guidelines [14, 15] at all study visits pre-bronchodilation. FEV_1_ and forced vital capacity (FVC) are expressed as %predicted using reference values published by Quanjer et al. [12]. The degree of air trapping (Residual Volume/Total Lung Capacity) is derived from lung volumes.

#### Cardiopulmonary exercise testing (CPET)

A continuous incremental cycle ergometer cardiopulmonary exercise test using the Godfrey protocol [16] is performed in accordance to a statement on exercise testing in CF [17]. Maximal aerobic power (Watt_max_) and VO_2_peak will be expressed in percent of predicted [16, 18].

#### Physical activity

Daily PA is measured using a tri-axial Omron HJ-322 pedometer for 7 days prior to baseline visit 1b and prior to the 6-months and 12-months visits. The 7-day recall questionnaire is used to assess time spent in the PA categories ‘hard’ and ‘very hard’. The questionnaire is a feasible and valid instrument to assess PA in people with CF [19].

#### Health-related quality of life (HRQoL)

HRQoL is assessed using the adolescent/adult version of the Cystic Fibrosis Questionnaire (CFQ-R), a validated CF-specific instrument [20, 21].

#### Psychological health

Psychological health is assessed using the Depression Anxiety Stress Scales (DASS), a 42-item self-report questionnaire that has been validated in clinical and non-clinical samples [22, 23].

#### Glycaemic control

Assessment of glycaemic control and potential diagnosis of cystic fibrosis related diabetes (CFRD) is performed using the oral glucose tolerance test after an 8 h fast according to a published consensus statement [24]. Blood samples are collected in a fasting state, and 1 and 2 h after the glucose challenge.

#### Exacerbations

Time to, number of and time with pulmonary exacerbations with and without hospitalisations, number of pulmonary exacerbations requiring intravenous antibiotic treatment, number of days on intravenous antibiotic therapy for pulmonary exacerbations and time to first intravenous antibiotic treatment for pulmonary exacerbations are assessed. The information is derived from a diary kept by each participant, 3-months retrospective questionnaires completed at each study visit, interviews at each study visit and scheduled telephone contact, and information provided by the participants at participant-initiated contacts. Each episode of pulmonary exacerbation defined as new or changed antibiotic therapy based on modified Fuchs criteria is recorded [25]. In addition, we note the number and times of outpatient visits to a medical doctor, clinic or hospital for CF-related complications with documentation of reasons. This includes the number of unplanned hospitalisations and their respective duration and reasons.

#### Upper respiratory tract infection (URTI)

As exacerbations, URTIs are identified from participants’ diary, from 3-months retrospective questionnaires completed at all clinic visits, from interviews at study visits and from telephone contacts. URTI are defined as at least 3 consecutive days of runny nose and sore throat.

#### Courses of unscheduled antibiotics

Antibiotic use is documented at all clinic and study visits as well as in participants’ diaries and antibiotics courses (intravenous, oral or inhaled) are counted. In case of exacerbation, planned study visits are postponed until 14 days after oral or intravenous antibiotic treatment of exacerbation has been stopped.

### Substudies

#### Accelerometry

In European countries, PA is assessed by an accelerometer (Actigraph GT1M or wGT3X, Pensacola, FL, USA), which is worn at the right hip over 7 days at baseline and prior to the 6 and 12-months study visits. The sampling epoch is set at 5 s and data are included if at least 4 full days (at least 3 weekdays and one weekend day) of measurements with a minimum of 10 h for the weekdays and 10 h for the weekends are measured [26]. Overall PA is expressed as average counts/min and time (min/day) in light, moderate and vigorous PA according to validated cut-off levels [27–29].

#### Multiple breath washout

Lung clearance index is determined using commercially available systems based on nitrogen washout (EasyOnePro Lab, ndd Medical Technologies, Zurich, Switzerland; ECO MEDICS AG, Dürnten, Switzerland) in centres with access to this or similar technology. Measurements are performed prior to spirometry and body plethysmography following the manufacturers’ instructions. The methodology has proven to be feasible in multi-centre trials [30].

#### Dual energy X-ray absorptiometry

Body composition and bone parameters are measured using dual-energy X-ray absorptiometry in Canadian and Swiss participants. Body composition is assessed by the three-compartment model, including fat mass, bone mineral content, and bone mineral free lean tissue. Bone mineral content and bone mineral density values are z-transformed using pubertal stage- and gender-specific means and standard deviations [31].

#### Mucociliary clearance

Mucociliary clearance (MCC) is measured using nuclear medicine scans at baseline (visit 1a), immediately after a submaximal exercise test (visit 1b), and then prior to their maximal CPET at patient’s 6-month study visit, to investigate the acute effects of exercise and the effects of increased activity and vigorous PA over a 6-month period on mucociliary clearance.

### Monitoring and quality assurance

Standard operational procedures (SOP) for all the methods used in the study are available in English. Moreover, essential parts of the SOPs are translated in all national languages of participating countries. A site initiation visit takes place at each center prior to the enrolment of the first patient in the study. A close out visit takes place when the last study participant at each center has completed the study.

Central data monitoring of REDCap database entries and on site monitoring are done for the majority of database entries, especially inclusion and exclusion criteria and study outcomes including safety data to assure high quality data. In particular, all lung function data entries and pseudonymized pulmonary function reports (primary endpoint FEV_1_) are checked for accuracy and correctness according to ATS/ERS standards [14, 15].

A close out visit takes place when the last study participant at each center has completed the study.

### Data safety monitoring board (DSMB)

For this study a DSMB Safety Board was implemented. Each serious safety issue is reported to the Board within 72 h. The safety board can request an interim analysis and can stop the study in case of serious safety issues.

### Sample size calculation

This study aims to enrol 292 patients with CF. The sample size is calculated based on the primary endpoint, i.e., change in FEV_1_ from baseline to 6 months between the intervention and control group. Two hundred two subjects with CF will be randomised in a 1:1 ratio to the intervention and the control group. Assuming a 5% screen failure rate, about 308 patients need to be screened. With an estimated 20% dropout rate after randomisation, this sample size would allow to detect a difference between intervention and control group after 6 months in FEV_1_ of 3% predicted (absolute change) with a power of 80% and a type 1-error probability of 5%. We expect that, by using stringent criteria to measure FEV_1_ within this study and by averaging two measurements 2–4 weeks apart to obtain the baseline value, we can bring down the standard deviation of the changes in FEV_1_ within each group from previously observed 10% to approximately 8%.

### Statistical analysis

Descriptive analyses using means and standard deviations, medians and interquartile ranges or means and 95% confidence intervals (CI’s) will be used for continuous variables as appropriate. Frequencies and proportions with 95% CI’s will be used for categorical variables. Primary analyses will be performed according to intention-to-treat (ITT) with all participants who were originally allocated by randomisation and those who dropped out from the study. ITT main analysis and analyses on secondary outpoints will be done including missing data imputations by multiple imputation, inverse probability weighing or mixed models as appropriate.

Additionally, we will perform sensitivity analyses using the per protocol set and run analyses based on *reported* and *assumed* compliance. The analysis of reported compliance will include all patients sufficiently compliant with the protocol and include those randomised into the intervention group with the addition of at least 2/3 of training volume (e.g., the addition of 2 h per week of vigorous PA) and compare to those randomised into the control group with no more than 30 min of extra vigorous PA per week during the period of interest than at baseline. The analysis on assumed compliance will include those from the intervention group with an increase of 5% and more of VO_2peak_ from baseline as marker of compliance with the programme and compare to those randomised into the control group with less than 5% increase of VO_2peak_ from baseline as marker of compliance with restraining from training.

The secondary efficacy endpoints will be analysed according to the main model described above. This includes the 0 to 6 months and the 0 to 12 months’ time periods for most variables, and the effects of the intervention on the change in FEV_1_ over 12 months, and the change in FEV_1_ between month 6 and month 12. Likewise, the change in glucose levels 1 and 2 h after a standardised glucose load between baseline and 9 months will be compared between groups. The endpoint “time to first exacerbation” will be analysed using a Cox proportional hazard model, while Poisson regression will be used for “number of upper respiratory tract infections”. The frequency of study participants developing diabetes mellitus between baseline assessments and 9-months follow-up will be compared between groups using chi square statistics. For safety endpoints, data will be monitored throughout the study for all patients. Analysis will be performed on total number of events and type of event (fracture, sprain, hypoglycaemia, haemoptysis, pneumothorax, etc.). The change from 6 to 12 months for all secondary outcomes and for the other explorative endpoints will be analysed with the main model described above except for “days on oral / intravenous antibiotics” which will be analysed using Poisson regression.

For the main analysis, the statistician and the PI will have full access to the dataset including the DSMB. Later, for secondary analysis, data will be shared among national coordinators.

## Discussion

This is the first large international randomised controlled trial to study the effects of regular vigorous PA with motivational feedback on important clinical, physiological and patient-reported outcomes in CF. Previous exercise training studies in CF were mostly of low to moderate methodological quality with small numbers of patients and conducted over short periods of time [3]. While the positive effects of exercise training and PA interventions on aerobic exercise capacity are predominantly consistent among studies, the effects on HRQoL and pulmonary function including FEV_1_ are less clear [3]. Two supervised exercise interventions demonstrated that regular vigorous exercise has the potential to positively impact FEV_1_ [4, 5]. These data are supported by observational studies, showing that regular PA is associated with a slower progression of FEV_1_ decline over time [32, 33]. Despite the limitations of FEV_1_ as clinical endpoint [34], FEV_1_ is the most commonly used endpoint in clinical trials and still the single best prognostic factor for CF [6]. The main aim of our study is to evaluate whether regular vigorous habitual PA can prevent deterioration of FEV_1_ decline over time that is much more clinically relevant than to demonstrate short-term improvements.

Our secondary aim is to study other important endpoints such as bone health, pulmonary exacerbations, glycaemia control and patient-reported outcomes such as HRQoL, depression and anxiety that have been rarely investigated in exercise training and PA interventions in CF [3]. Some of these endpoints have been previously investigated in cross-sectional, observational and/or small randomised controlled trials showing some beneficial effects of PA on bone health [35], glycaemic control [36] and hospitalisation days [33], but these data need to be further substantiated by a large randomised controlled study and ideally including patients with a broad age range and disease severity to be representative for the overall CF population. The focus of patient-reported outcomes is very important and in the past has been often neglected in trials.

A critical point in each PA intervention study especially in chronic disease is adherence and compliance of the patients. Using pedometers to provide feedback on daily PA can enhance motivation to exercise [37] and has been shown to be effective in activity programs addressing people with a variety of chronic health conditions (10) including youth [38] and patients with chronic obstructive pulmonary disease [39]. Providing additional feedback on exercise behaviour by keeping a diary as well as giving personal feedback via a SMS message to the cellular phone, email or telephone also enhanced adherence to a home-based exercise programme that was partially supervised [40, 41].

The major strength of the current investigation is the inclusion of a heterogeneous study population with a broad range of disease severity (FEV_1_ ≥ 35% predicted) and recruited from eight different countries in Europe and Northern America. The use of modern technology (step counters and web-based diary) for PA monitoring is unique to exercise studies in this patient population and will provide new insights into the usefulness and feasibility of these wearables.

### Limitations

Due to the nature of a partially supervised study, adherence to PA and data entries in the web-based diary cannot be fully controlled. However, objective measures of exercise capacity using CPET and PA (substudy using Actigraph accelerometry) will be used to verify training effects after 6 and 12-months and will provide an indication of whether study participants adhered to their training schedule or not. Blinding of study participants to minimise bias is not possible in PA intervention studies, but outcome assessors are blinded, whenever possible. A selection bias towards a physically active CF population may occur, because patients who are physically active in their daily lives might be more likely to participate in this trial.

Patient recruitment for long-term PA interventions is challenging [5], in particular in rare diseases such as CF. Moreover, there are several recent drug trials on the effects of CF transmembrane conductance regulator corrector and potentiator therapy that might interfere with the ACTIVATE-CF trial. Major efforts have been done with respect to study center recruitment. The study is currently running in > 20 specialised CF centers in eight countries in Europe and Northern America. The first study participant was enrolled in June 2014 and the last study participant is expected to complete the last study visit in June 2018.

This study could make an important contribution to the knowledge on the effects of regular vigorous PA on several important health outcomes in CF. In particular, the use of modern PA monitoring and motivational feedback embedded in the intervention will enhance our understanding on the feasibility and usefulness of these tools with respect to long-term PA adherence.

## Abbreviations

CF: Cystic fibrosis
CFQ-R: Cystic fibrosis questionnaire-revised
CFRD: Cystic fibrosis related diabetes
CI: Confidence interval
CPET: Cardiopulmonary exercise testing
DASS: Depression anxiety stress scales
DSMB: Data safety monitoring board
eCRF: electronic Case Report Form
FEV_1_: Forced expiratory volume in 1 s
FVC: Forced vital capacity
HRQoL: Health-related quality of life
ITT: Intention-to-treat
MCC: Mucociliary clearance
PA: Physical activity
SOP: Standard operating procedure
URTI: Upper respiratory tract infection
VO_2_peak: Peak oxygen uptake
Watt_max_: Maximal aerobic power

## References

[1] Dwyer TJ, Elkins MR, Bye PT (2011). The role of exercise in maintaining health in cystic fibrosis. Curr Opin Pulm Med.

[2] Rand S, Prasad SA (2012). Exercise as part of a cystic fibrosis therapeutic routine. Expert Rev Respir Med.

[3] Radtke, T, Nevitt SJ, Hebestreit H, Kriemler S. Physical exercise training for cystic fibrosis. Cochrane Database Syst Rev. 2017;11:CD002768. 10.1002/14651858.CD002768.pub4 PMC6485991 29090734

[4] Selvadurai HC, Blimkie CJ, Meyers N, Mellis CM, Cooper PJ, Van Asperen PP (2002). Randomized controlled study of in-hospital exercise training programs in children with cystic fibrosis. Pediatr Pulmonol.

[5] Kriemler S, Kieser S, Junge S, Ballmann M, Hebestreit A, Schindler C, Stussi C, Hebestreit H (2013). Effect of supervised training on FEV1 in cystic fibrosis: a randomised controlled trial. J Cyst Fibros.

[6] Navarro J, Rainisio M, Harms HK, Hodson ME, Koch C, Mastella G, Strandvik B, McKenzie SG (2001). Factors associated with poor pulmonary function: cross-sectional analysis of data from the ERCF. European epidemiologic registry of cystic fibrosis. Eur Respir J.

[7] Hebestreit H, Kieser S, Junge S, Ballmann M, Hebestreit A, Schindler C, Schenk T, Posselt HG, Kriemler S (2010). Long-term effects of a partially supervised conditioning programme in cystic fibrosis. Eur Respir J.

[8] Gulmans VA, de Meer K, Brackel HJ, Faber JA, Berger R, Helders PJ (1999). Outpatient exercise training in children with cystic fibrosis: physiological effects, perceived competence, and acceptability. Pediatr Pulmonol.

[9] Tudor-Locke C, Lutes L (2009). Why do pedometers work?: a reflection upon the factors related to successfully increasing physical activity. Sports Med.

[10] Bravata DM, Smith-Spangler C, Sundaram V, Gienger AL, Lin N, Lewis R, Stave CD, Olkin I, Sirard JR (2007). Using pedometers to increase physical activity and improve health - a systematic review. JAMA.

[11] Smith KM, Arthur HM, McKelvie RS, Kodis J (2004). Differences in sustainability of exercise and health-related quality of life outcomes following home or hospital-based cardiac rehabilitation. Eur J Cardiovasc Prev Rehabil.

[12] Quanjer PH, Stanojevic S, Cole TJ, Baur X, Hall GL, Culver BH, Enright PL, Hankinson JL, Ip MS, Zheng J, Stocks J (2012). Multi-ethnic reference values for spirometry for the 3-95-yr age range: the global lung function 2012 equations. Eur Respir J.

[13] American College of Sports Medicine (2009). Guidelines for exercise testing and prescription.

[14] Miller MR, Hankinson J, Brusasco V, Burgos F, Casaburi R, Coates A, Crapo R, Enright P, van der Grinten CP, Gustafsson P, Jensen R, Johnson DC, MacIntyre N, McKay R, Navajas D, Pellegrino R, Viegi G, Wanger J, Pedersen OF (2005). Standardisation of spirometry. Eur Respir J.

[15] Wanger J, Clausen JL, Coates A, Brusasco V, Burgos F, Casaburi R, Crapo R, Enright P, van der Grinten CP, Gustafsson P, Hankinson J, Jensen R, Johnson D, Macintyre N, McKay R, Miller MR, Navajas D, Pellegrino R, Viegi G, Pedersen OF (2005). Standardisation of the measurement of lung volumes. Eur Respir J.

[16] Godfrey S (1970). Exercise tests in assessing children with lung or heart disease. Thorax.

[17] Hebestreit H, Arets HG, Aurora P, Boas S, Cerny F, Hulzebos EH, Karila C, Lands LC, Lowman JD, Swisher A, Urquhart DS, European Cystic Fibrosis Exercise Working G (2015). Statement on exercise testing in cystic fibrosis. Respiration.

[18] Orenstein DM, Rowland TW (1993). Assessment of exercise pulmonary function. Pediatric laboratory exercise testing clinical guidelines.

[19] Ruf K, Fehn S, Bachmann M, Moeller A, Roth K, Kriemler S, Hebestreit H (2012). Validation of activity questionnaires in patients with cystic fibrosis by Accelerometry and cycle Ergometry. BMC Med Res Methodol.

[20] Quittner AL, Buu A, Messer MA, Modi AC, Watrous M (2005). Development and validation of the cystic fibrosis questionnaire in the United States: a health-related quality-of-life measure for cystic fibrosis. Chest.

[21] Wenninger K, Aussage P, Wahn U, Staab D (2003). The revised German cystic fibrosis questionnaire: validation of a disease-specific health-related quality of life instrument. Qual Life Res.

[22] Brown TA, Chorpita BF, Korotitsch W, Barlow DH (1997). Psychometric properties of the depression anxiety stress scales (DASS) in clinical samples. Behav Res Ther.

[23] Crawford JR, Henry JD (2003). The depression anxiety stress scales (DASS): normative data and latent structure in a large non-clinical sample. Br J Clin Psychol.

[24] Moran A, Brunzell C, Cohen RC, Katz M, Marshall BC, Onady G, Robinson KA, Sabadosa KA, Stecenko A, Slovis B (2010). Clinical care guidelines for cystic fibrosis-related diabetes: a position statement of the American Diabetes Association and a clinical practice guideline of the Cystic Fibrosis Foundation, endorsed by the pediatric Endocrine Society. Diabetes Care.

[25] Bilton D, Canny G, Conway S, Dumcius S, Hjelte L, Proesmans M, Tummler B, Vavrova V, De Boeck K (2011). Pulmonary exacerbation: towards a definition for use in clinical trials. Report from the EuroCareCF working group on outcome parameters in clinical trials. J Cyst Fibros.

[26] Freedson P, Pober D, Janz KF (2005). Calibration of accelerometer output for children. Med Sci Sports Exerc.

[27] Kozey SL, Lyden K, Howe CA, Staudenmayer JW, Freedson PS (2010). Accelerometer output and MET values of common physical activities. Med Sci Sports Exerc.

[28] Troiano RP, Berrigan D, Dodd KW, Masse LC, Tilert T, McDowell M (2008). Physical activity in the United States measured by accelerometer. Med Sci Sports Exerc.

[29] Trost SG, Loprinzi PD, Moore R, Pfeiffer KA (2011). Comparison of accelerometer cut points for predicting activity intensity in youth. Med Sci Sports Exerc.

[30] Fuchs SI, Ellemunter H, Eder J, Mellies U, Grosse-Onnebrink J, Tummler B, Staab D, Jobst A, Griese M, Ripper J, Rietschel E, Zeidler S, Ahrens F, Gappa M (2012). Feasibility and variability of measuring the lung clearance index in a multi-center setting. Pediatr Pulmonol.

[31] Zemel BS, Kalkwarf HJ, Gilsanz V, Lappe JM, Oberfield S, Shepherd JA, Frederick MM, Huang X, Lu M, Mahboubi S, Hangartner T, Winer KK (2011). Revised reference curves for bone mineral content and areal bone mineral density according to age and sex for black and non-black children: results of the bone mineral density in childhood study. J Clin Endocrinol Metab.

[32] Schneiderman JE, Wilkes DL, Atenafu EG, Nguyen T, Wells GD, Alarie N, Tullis E, Lands LC, Coates AL, Corey M, Ratjen F (2014). Longitudinal relationship between physical activity and lung health in patients with cystic fibrosis. Eur Respir J.

[33] Cox NS, Alison JA, Button BM, Wilson JW, Morton JM, Holland AE. Physical activity participation by adults with cystic fibrosis: an observational study. Respirology. 2016;21:511–8. 10.1111/resp.12719 26715596

[34] Stanojevic S, Ratjen F (2016). Physiologic endpoints for clinical studies for cystic fibrosis. J Cyst Fibros.

[35] Tejero Garcia S, Giraldez Sanchez MA, Cejudo P, Quintana Gallego E, Dapena J, Garcia Jimenez R, Cano Luis P, Gomez de Terreros I (2011). Bone health, daily physical activity, and exercise tolerance in patients with cystic fibrosis. Chest.

[36] Moran A, Dunitz J, Nathan B, Saeed A, Holme B, Thomas W (2009). Cystic fibrosis-related diabetes: current trends in prevalence, incidence, and mortality. Diabetes Care.

[37] Lauzon N, Chan CB, Myers AM, Tudor-Locke C (2008). Participant experiences in a workplace pedometer-based physical activity program. J Phys Act Health.

[38] Lubans DR, Morgan PJ, Tudor-Locke C (2009). A systematic review of studies using pedometers to promote physical activity among youth. Prev Med.

[39] Hospes G, Bossenbroek L, ten Hacken NHT, van Hengel P, de Greef MHG (2009). Enhancement of daily physical activity increases physical fitness of outclinic COPD patients: results of an exercise counseling program. Patient Educ Couns.

[40] Blaauwbroek R, Bouma MJ, Tuinier W, Groenier KH, de Greef MH, Meyboom-de Jong B, Kamps WA, Postma A (2009). The effect of exercise counselling with feedback from a pedometer on fatigue in adult survivors of childhood cancer: a pilot study. Support Care Cancer.

[41] Strath SJ, Swartz AM, Parker SJ, Miller NE, Grimm EK, Cashin SE (2011). A pilot randomized controlled trial evaluating motivationally matched pedometer feedback to increase physical activity behavior in older adults. J Phys Act Health.

